# Supplementary material for: Variant selection to maximize variance explained in cis-Mendelian randomization
Source: HGG Adv. 2026 Jan 16;7(2):100573. doi: 10.1016/j.xhgg.2026.100573 (PMC12887808; doi:10.1016/j.xhgg.2026.100573)
Supplement: Document S1. Figures S1–S3 and supplemental material and methods [file mmc1.pdf]

HGGA, Volume *7*

Supplemental information

Variant selection to maximize variance explained in *cis*-Mendelian randomization

Ang Zhou, Ville Karhunen, Haodong Tian, Janne Pott, Ashish Patel, Eric A.W. Slob, and Stephen Burgess

1. Calculation of F-statistics and R^2 with correlated variants using GWAS summary statistics

A: Linear IV model with multiple (potentially correlated) IVs

Let Y denote an outcome, X a potential risk factor for the outcome, and $Z = (Z_1, \ldots, Z_J)'$ a J-vector of potentially correlated IVs. Then, we consider the linear IV model

$$\begin{aligned} Y &= \alpha + X\theta_0 + U && (1) \\ X &= \omega + Z'\gamma + V && (2) \end{aligned}$$

where the error terms satisfy $E[U|Z] = 0$, $E[V|Z] = 0$. Since we are working with genetic association summary data, we are forced to make the assumption that these structural errors are homoskedastic, $E[U^2|Z] = \sigma_U^2$ and $E[V^2|Z] = \sigma_V^2$.

B: F-statistics under individual-level data

For a sample size N, an estimate of γ from the first-stage linear regression of X on Z is given by $\widehat{\gamma} = \widehat{var}(Z)^{-1}\widehat{cov}(Z,X)$. An estimate of the variance of $\widehat{\gamma}$ is $\widehat{\Sigma}_\gamma = \widehat{var}(Z) \otimes (\widehat{var}(X) - \widehat{cov}(Z,X)'\widehat{var}(X)^{-1}\widehat{cov}(Z,X))$. Then, a test of instrument relevance considers evidence against the null hypothesis $H_0 : \gamma = 0$, using the statistic

$$\widehat{W}_\gamma = (N - J)\widehat{\gamma}'\widehat{\Sigma}_\gamma^{-1}\widehat{\gamma}. \tag{3}$$

Note that by a central limit theorem and CramÃ©r's Theorem, since $\widehat{\Sigma}_\gamma \xrightarrow{p} var(Z) \otimes \sigma_V^2$, under $H_0 : \gamma = 0$, we have $\widehat{\Sigma}_\gamma^{-\frac{1}{2}}\sqrt{N-J}\widehat{\gamma} \xrightarrow{d} N(0, I_{J\times J})$ as $n \to \infty$. Hence, $\widehat{W}_\gamma \xrightarrow{d} \chi_J^2$ as $n \to \infty$.

The F-test statistic is defined as $\widehat{F} = \widehat{W}_\gamma / J$.

C: An R^2 measure under individual-level data

The sample R^2 in the model of Equation (2) is $\widehat{R}^2 = \widehat{\gamma}'\widehat{var}(Z)\widehat{\gamma}/\widehat{var}(X)$. The F-statistic $\widehat{F}$ can be written in terms of $\widehat{R}^2$,

$$\widehat{F} = \frac{N - J - 1}{J} \cdot \frac{\widehat{R}^2}{1 - \widehat{R}^2} \tag{4}$$

Also, from Equation (4), $J\widehat{F}(1 - \widehat{R}^2) = (N - J - 1)\widehat{R}^2$ so that $J\widehat{F} = (N - J - 1 + J\widehat{F})\widehat{R}^2$ and

$$\widehat{R}^2 = \frac{J\widehat{F}}{N - J - 1 + J\widehat{F}}. \tag{5}$$

D: Calculating F-statistics and R^2 with univariable summary data

Suppose we have access to:

1. signed genetic variant correlations between any two variants j_1 and j_2, denoted $\rho_{j_1 j_2} = \widehat{cor}(Z_{j_1}, Z_{j_2})$
2. beta-coefficients from a linear regression of X on Z_j, denoted $\widehat{\beta}_j = \widehat{var}(Z_j)^{-1}\widehat{cov}(Z_j, X)$
3. standard errors of $\widehat{\beta}_j$, denoted as the square root of $\widehat{\sigma}_j^2 = \left[\widehat{var}(X) - \widehat{\beta}_j^2 \widehat{var}(Z_j)\right] \Big/ \left[N \cdot \widehat{var}(Z_j)\right]$
4. the sample size N.

Then, note that $a_j = N\widehat{\sigma}_j^2 + \widehat{\beta}_j^2 = \widehat{var}(Z_j)^{-1}\widehat{var}(X)$. Second, let $b_j = a_j^{-1}\widehat{\beta}_j = \widehat{var}(X)^{-1}\widehat{cov}(Z_j, X)$ so that $b = (b_1, \ldots b_J)' = \widehat{cov}(Z, X)\widehat{var}(X)^{-1}$.

Next, let C be a $J \times J$ matrix with its (j_1, j_2)-th element given by $c_{j_1 j_2} = \rho_{j_1 j_2} a_{j_1}^{-\frac{1}{2}} a_{j_2}^{-\frac{1}{2}}$ $= \widehat{cor}(Z_{j_1}, Z_{j_2})\widehat{var}(Z_{j_1})^{\frac{1}{2}}\widehat{var}(Z_{j_2})^{\frac{1}{2}}\widehat{var}(X)^{-1} = \widehat{cov}(Z_{j_1}, Z_{j_2})\widehat{var}(X)^{-1}$. Therefore, $C = \widehat{var}(Z)\widehat{var}(X)^{-1}$. Then, $\widehat{\Gamma} = \widehat{var}(Z)^{-1}\widehat{cov}(Z, X)$, i.e. the beta coefficient from a *multivariable* linear regression of X on $Z = (Z_1, \ldots, Z_J)'$ can be computed as

$$\widehat{\Gamma} = C^{-1}b$$

and the variance-covariance matrix of $\widehat{\Gamma}$, which is $\widehat{\Sigma}_\gamma = \widehat{var}(Z)^{-1} \cdot \left[\widehat{var}(X) - \widehat{cov}(Z, X)'\widehat{var}(Z)^{-1}\widehat{cov}(Z, X)\right]$ can be computed as

$$\widehat{\Sigma}_\gamma = C^{-1} \cdot \left[1 - b'C^{-1}b\right].$$

Using Equation (3), we can plug in our estimates $\widehat{\Gamma}$ and $\widehat{\Sigma}_\gamma$ into Equation (3) to calculate $\widehat{W}_\gamma$, and then just divide by J to calculate the $\widehat{F}$ statistic as $\widehat{F} = \widehat{W}_\gamma \big/ J$.

Finally, after calculating $\widehat{F}$, just plug-in the estimate into Equation (5) to get the $\widehat{R}^2$ estimate.

2. Extended simulation study I

In practice, we often do not know whether the true causal variant(s) are captured by the genotyping array. Indeed, it is likely that in many cases they are not directly genotyped and are only tagged by nearby variants. To approximate this situation in the single-causal-variant scenario described in the main text, we removed the causal variant along with all variants in linkage disequilibrium with it above a given threshold (pairwise $r^2 > 0.95$, or > 0.8, or > 0.4) from the dataset. We then assessed how well different variant selection strategies could recover the true genetic variance. Results for this simulation analysis are illustrated in Figure S2.

As expected, genetic predictors derived from all strategies explained less variance in the simulated exposure than that by the true causal variant, because the causal variant and its close proxies had been removed from the dataset (Figure S2). For strategies that require a tuning parameter, such as modified LD-pruning, COJO, and PCA, increasing the tuning parameter led to more variants in the gene region being incorporated into the genetic predictor, resulting in a greater proportion of variance explained (Figure S2). All variant selection strategies that incorporate multiple variants from the gene region explained more variance than the lead-variant-only approach, likely because these additional variants partially recover information about the removed causal variant. The advantage of these multi-variant strategies became increasingly apparent as variants with progressively lower correlation thresholds to the causal variant (from $r^2 > 0.95$ down to $r^2 > 0.4$) were removed, which further weakened the performance of the lead-variant-only approach (Figure S2 C).

3. Extended simulation study II

We further evaluated the performance of multi-variant strategies in the presence of horizontal pleiotropy. Specifically, in this simulation we considered a scenario in which two independent causal variants influence the exposure, and horizontal pleiotropy is introduced by allowing one of these causal variants to also influence the outcome. There is no actual causal effect of the exposure on the outcome. Therefore, any deviation of MR estimate from the null indicates bias. Data generating mechanism is shown below:

$$X_i = \sqrt{0.25}G_{i1} + \sqrt{0.15}G_{i2} + \varepsilon_{xi}$$

$$Y_i = \sqrt{0.05}G_{i2} + \varepsilon_{yi}$$

where: X_i: Simulated exposure for individual I,

Y_i: Simulated outcome for individual I,

G_{i1}: Standardized genotyping data for the first causal variant,

G_{i2}: Standardized genotyping data for the second causal variant,

$\varepsilon_{xi} \sim N(0, 0.6)$: Residual error term for exposure with variance being 60%,

$\varepsilon_{yi} \sim N(0, 0.95)$: Residual error term for outcome with variance being 95%.

This simulation experiment was repeated 100 times. Results for this simulation analysis are shown in the Figure S3.

As expected, the lead-variant-only approach produced an unbiased estimate, because the stronger causal variant has no horizontal pleiotropic effect. In contrast, all multi-variant strategies produced biased MR estimates, presumably because the genetic predictors derived from these strategies included the pleiotropic causal variant or variants correlated with it (Figure S3).

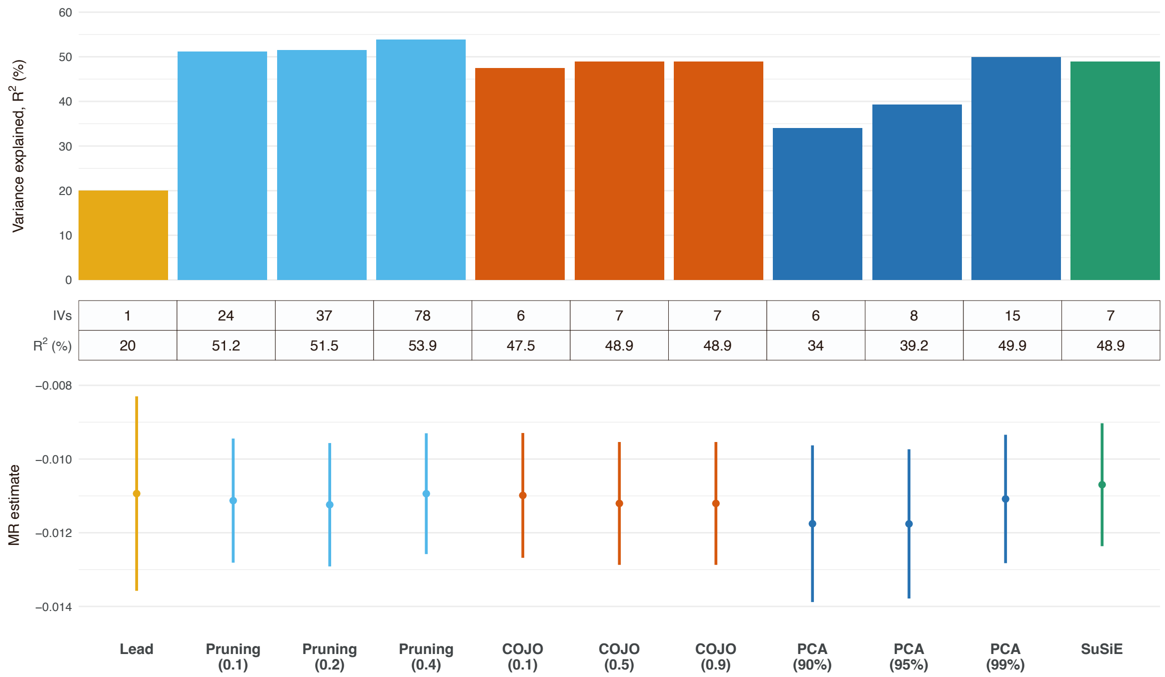

Figure S1: Proportion of variance in haptoglobin levels explained by variants selected using modified LD-pruning, COJO, SuSiE, and PCA, together with the corresponding MR estimates for the effect on red blood cell count, using the full set of variants in the haptoglobin region without p-value pre-filtering at the 0.001 threshold. The table below the plot shows the number of IVs and variance explained, R^2 (%). Error bars represent 95% CIs. For the modified LD-pruning, results are shown for pruning thresholds of r^2 = 0.1, 0.2 and 0.4. For PCA, results correspond to PCs capturing 90%, 95% and 99% of the variance in the LD matrix. For COJO, results are shown for collinearity thresholds of 0.1, 0.5, and 0.9.

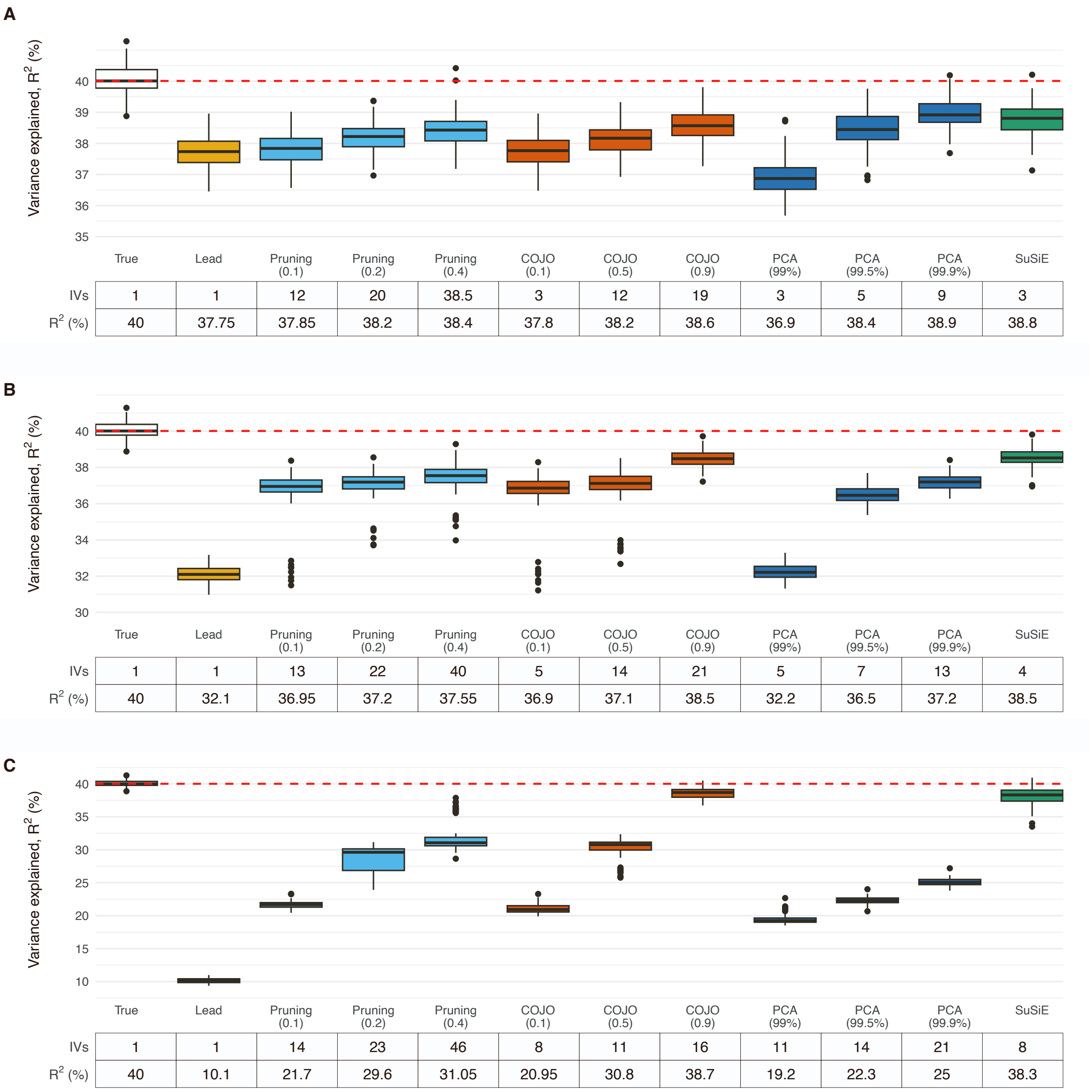

A

	True	Lead	Pruning (0.1)	Pruning (0.2)	Pruning (0.4)	COJO (0.1)	COJO (0.5)	COJO (0.9)	PCA (99%)	PCA (99.5%)	PCA (99.9%)	SuSiE
IVs	1	1	12	20	38.5	3	12	19	3	5	9	3
R^2 (%)	40	37.75	37.85	38.2	38.4	37.8	38.2	38.6	36.9	38.4	38.9	38.8

B

	True	Lead	Pruning (0.1)	Pruning (0.2)	Pruning (0.4)	COJO (0.1)	COJO (0.5)	COJO (0.9)	PCA (99%)	PCA (99.5%)	PCA (99.9%)	SuSiE
IVs	1	1	13	22	40	5	14	21	5	7	13	4
R^2 (%)	40	32.1	36.95	37.2	37.55	36.9	37.1	38.5	32.2	36.5	37.2	38.5

C

	True	Lead	Pruning (0.1)	Pruning (0.2)	Pruning (0.4)	COJO (0.1)	COJO (0.5)	COJO (0.9)	PCA (99%)	PCA (99.5%)	PCA (99.9%)	SuSiE
IVs	1	1	14	23	46	8	11	16	11	14	21	8
R^2 (%)	40	10.1	21.7	29.6	31.05	20.95	30.8	38.7	19.2	22.3	25	38.3

Figure S2: Proportion of variance in a simulated trait with one causal variant explained by variants selected using modified LD-pruning, COJO, SuSiE, and PCA, under scenarios where the causal SNP and its correlated SNPs are removed at three LD thresholds: (A) $r^2 > 0.95$, (B) $r^2 > 0.80$, and (C) $r^2 > 0.40$. Each simulation was repeated 100 times. The table below each plot shows the median number of IVs and median R^2 (%) across 100 replicates. The red dashed line indicates the median true R^2 (%), calculated from regressing the exposure on the true causal variant(s) using individual level data. Boxplots represent the estimates from each replicate. The box displays the lower quartile,

median, and upper quartile; whiskers extend to the minimum and maximum values within 1.5 x interquartile range from the lower and upper quartiles. Estimates outside this range are shown as individual points. For the modified LD-pruning, results are shown for pruning thresholds of r^2 = 0.1, 0.2 and 0.4. For PCA, results correspond to PCs capturing 99%, 99.5% and 99.9% of variance in the LD matrix. For COJO, results are shown for collinearity thresholds of 0.1, 0.5, and 0.9.

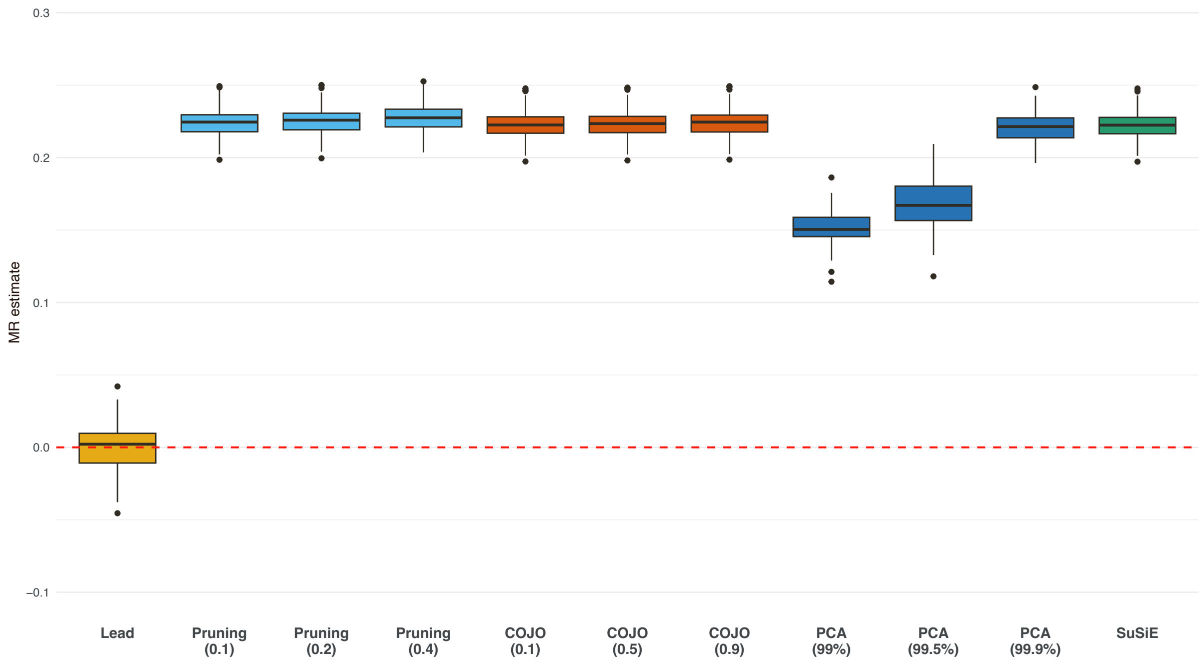

Figure S3: MR estimates obtained using variants selected by modified LD-pruning, COJO, SuSiE, and PCA in the simulation scenario with horizontal pleiotropy. Each simulation was repeated 100 times. The red dashed line indicates the target effect estimate. Boxplots represent the estimates from each replicate. The box displays the lower quartile, median, and upper quartile; whiskers extend to the minimum and maximum values within 1.5 x interquartile range from the lower and upper quartiles. Estimates outside this range are shown as individual points. For the modified LD-pruning, results are shown for pruning thresholds of r^2 = 0.1, 0.2 and 0.4. For PCA, results correspond to PCs capturing 99%, 99.5% and 99.9% of variance in the LD matrix. For COJO, results are shown for collinearity thresholds of 0.1, 0.5, and 0.9.
