# Supplementary material for: Variant selection to maximize variance explained in cis-Mendelian randomization
Source: HGG Adv. 2026 Jan 16;7(2):100573. doi: 10.1016/j.xhgg.2026.100573 (PMC12887808; doi:10.1016/j.xhgg.2026.100573)
Supplement: Document S2. Article plus supplemental information [file mmc3.pdf]

HGG
Advances

ARTICLE

Variant selection to maximize variance explained in *cis*-Mendelian randomization

Ang Zhou,[1,3,7,*] Ville Karhunen,[1] Haodong Tian,[1,4,6] Janne Pott,[1] Ashish Patel,[1] Eric A.W. Slob,[1,5] and Stephen Burgess[1,2]

Summary

Optimal selection of instrumental variables (IVs) from a single gene region in *cis*-Mendelian randomization (MR) is challenging, as variants are highly correlated due to linkage disequilibrium (LD). Using only the lead variant is convenient but may not achieve full statistical power if multiple signals exist. We compared four selection methods that incorporate correlated non-lead variants, including LD-pruning, conditional and joint analysis (COJO), sum of single effects (SuSiE) regression, and principal component analysis (PCA), and evaluated their ability to increase instrument strength, measured by variance explained in the exposure (R^2), relative to the lead-variant-only approach. We applied these methods to circulating haptoglobin (HP), to simulated traits with known variance explained, and to 15 additional gene regions where non-lead *cis*-protein quantitative trait loci (pQTLs) contributed varying proportions of *cis*-genetic variance. R^2 was estimated from variant-protein association estimates (Fenland study, $n = 10{,}708$) using LD from the UK Biobank ($n = 356{,}557$). In the *HP* region, the four methods produced a median proportional gain in R^2 of 145.1% compared with the lead variant alone (range: 69.6%–169.4%), with a median reduction in the MR standard error of 36.3% (range: −37.9% to −19.3%). In simulations, all methods were able to recover the expected genetic variance. Across the 15 gene regions, methods incorporating non-lead variants consistently outperformed the lead-variant-only approach. Variant selection methods incorporating correlated non-lead variants can reliably improve instrument strength in *cis*-MR analyses. We recommend using such methods but advise comparing their estimates with the lead-variant-only estimate to safeguard against numerical instability.

Introduction

Mendelian randomization (MR) is an epidemiological approach that uses genetic variants as instrumental variables (IVs) to estimate the causal effect of an exposure on an outcome. Given its reliance on genetic instruments, the selection of appropriate genetic variants is critical for a reliable MR investigation. Aside from instrument validity, another crucial consideration when choosing an instrument is its strength—a strong genetic instrument not only boosts statistical power but also minimizes weak instrument bias.[1] To ensure instrument strength, genetic instruments are typically selected from genome-wide association analyses (GWASs) of the exposure of interest, using independent top hits spanning multiple loci across the genome.[2]

cis-MR is a special type of MR in which genetic variants are selected from a single gene region, typically in settings where the exposure of interest is a molecular trait, such as protein or gene expression levels. It has been increasingly used to study the potential effects of pharmacological perturbation of drug targets, helping to inform drug development strategies.[3] Instrument selection in *cis*-MR is challenging, as variants in a gene region are typically highly correlated due to linkage disequilibrium (LD). A common and convenient approach is to choose only the lead variant in the region. However, there is growing recognition that a gene locus can contain more than one signal,[4] suggesting that the lead-variant-only approach may not be efficient and that incorporating non-lead variants in a gene region as IVs may provide additional instrument strength, thereby improving inferences. While it may be tempting to use all variants in the region associated with the trait of interest, doing so would require inverting the genotype correlation matrix (also called the LD matrix), which may be ill-conditioned, resulting in numerically unstable MR estimates.[5]

The ideal set of IVs for a *cis*-MR analysis, in terms of maximizing variance explained, would include all variants in the gene region that independently contribute to explaining variance in the trait of interest. As such, optimal variant selection is essentially a fine-mapping problem.[6] However, finding such variants is challenging in practice due to LD. It is important to note that variants used in MR do not need to be causal themselves; they are considered valid IVs as long as they satisfy the core MR assumptions. Various strategies with distinct goals have been proposed to derive sets of variants that explain a high proportion of variance in the exposure, including LD-pruning, conditional and joint analysis (COJO),[7]

[1]MRC Biostatistics Unit, University of Cambridge, Cambridge, UK; [2]Cardiovascular Epidemiology Unit, Department of Public Health and Primary Care, University of Cambridge, Cambridge, UK; [3]Australian Centre for Precision Health, Unit of Clinical and Health Sciences, University of South Australia, Adelaide, SA, Australia; [4]Center for Genomic Medicine, Massachusetts General Hospital, Boston, MA, USA; [5]Department of Psychology, Education & Child Studies, Erasmus University Rotterdam, Rotterdam, the Netherlands; [6]Broad Institute of MIT and Harvard, Cambridge, MA, USA
[7]Lead contact
*Correspondence: ang.zhou@mrc-bsu.cam.ac.uk
https://doi.org/10.1016/j.xhgg.2026.100573.
© 2026 The Authors. Published by Elsevier Inc. on behalf of American Society of Human Genetics.
This is an open access article under the CC BY license (http://creativecommons.org/licenses/by/4.0/).

sum of single effects (SuSiE) regression,[8,9] and principal component analysis (PCA).[5] LD-pruning is a widely used filtering approach aimed at identifying low-correlated genetic predictors of the exposure. It iteratively retains variants with the strongest marginal associations while removing those in high LD. COJO and SuSiE are two widely used fine-mapping methods (among others, such as FINEMAP[10] and FiniMoM[11]) designed to prioritize or identify putative causal variants, with COJO representing a frequentist approach and SuSiE a Bayesian approach. COJO uses a conditional regression framework to account for the correlation structure among variants and iteratively selects variants that have conditionally independent associations with the trait. SuSiE adopts a Bayesian variable selection strategy that simultaneously models all variants in a gene region and probabilistically identifies clusters of variants likely to contain causal signals. PCA aims to identify combinations of variants that capture variance in genetic associati0ons. Rather than selecting individual variants as instruments, it constructs linear weighted scores that are the principal components (PCs) from the decomposition of a weighted version of the genotype correlation matrix.

In this study, we aimed to compare the predictive strength, measured by variance in the exposure trait explained, of variant sets derived from LD-pruning, COJO, SuSiE, and PCA methods and assess whether they offer a reliable gain over using the lead variant alone. To address this aim, we evaluated the performance of the four methods using variants in the *haptoglobin* (*HP*) (MIM: 140100) gene region in predicting circulating plasma levels of HP. We note that findings from this motivating example are limited by the absence of ground truth regarding the true variance explained by causal variants and may not be generalizable to other gene regions with different genetic architectures. To address these limitations, we then evaluated the performance of the four methods using simulated traits with known genetic variance explained, allowing us to assess how reliably variant sets from each method capture the true underlying genetic contribution. Finally, to enhance the generalizability of our findings beyond the *HP* gene region, we extended our analysis to fifteen additional gene regions, each with differing estimated levels of variance explained by non-lead *cis*-variants.

Material and methods

Variant selection methods

Our study evaluated four variant selection methods: LD-pruning, COJO, SuSiE, and PCA. This section first describes their common features, including input data, variant filtering criteria, and tuning parameters, and then outlines the algorithmic steps specific to each method.

Input data

LD-pruning, SuSiE, and PCA require variant-exposure association estimates, their standard errors, and a pre-computed LD matrix as inputs. COJO also uses variant-exposure association estimates and their standard errors, but unlike the other three methods, it additionally requires individual-level genotype data to compute variant correlations for guiding variant selection.

Variant filtering criteria

To remove irrelevant variants and reduce computational burden, variants are filtered based on their marginal association p values with the exposure before applying LD-pruning, SuSiE, and PCA, retaining only those with $p < 0.001$ for subsequent selection. No prior filtering is applied for COJO, as the algorithm includes an internal filtering process based on variants' conditional p values (see section COJO algorithm). To ensure consistency across methods, the same p value threshold of 0.001 is specified when running COJO. PCA involves an additional filtering step, where variants are pruned using a pairwise squared correlation, an r^2 threshold of 0.95, to remove extremely highly correlated variants. To assess the impact of the marginal p value pre-filtering step, we conducted a sensitivity analysis in which we repeated the HP-region analyses using the full set of variants without applying the filter. Results were essentially unchanged across all variant selection strategies (Figure S1).

Tuning parameter

For LD-pruning, COJO, and PCA, variant selection depends on a tuning parameter that influences how many variants are retained. Larger values of this tuning parameter correspond to more lenient selection criteria, resulting in more variants being included for selection. Specifically, the tuning parameter is the pairwise r^2 pruning threshold for LD-pruning, the collinearity threshold for COJO, and the proportion of variance in the LD matrix explained by PCs for PCA. In our analyses, we varied these parameters systematically using small, arbitrary step sizes to capture fine-grained changes in variant selection and the resulting variance explained. Further details on the choices of tuning parameters relevant for each method are provided below.

LD-pruning algorithm

Standard LD-pruning is a widely used method for variant selection due to its simplicity. The algorithm begins by selecting the variant with the smallest marginal p value of univariable variant-exposure associations. In the case of a tie, one variant is randomly selected. For the remaining variants, any variant with a pairwise r^2 exceeding a pre-specified threshold with the selected variant is excluded from further consideration. This selection and exclusion process is repeated until no additional variants can be selected. The result is a set of variants where the pairwise r^2 between any two variants is below the specified threshold. A key limitation of the standard LD-pruning method is that its variant selection relies only on marginal p values and pairwise r^2 threshold, with no mechanism to ensure that the added variants meaningfully improve exposure prediction. While adding more variants may appear to increase the variance explained, R^2, this increase may not reflect genuine predictive gain, as R^2 is non-decreasing by construction. Moreover, retaining more correlated variants can potentially introduce multicollinearity through haplotype structure, even when no individual variant pair is highly correlated, causing the LD matrix to become singular and preventing MR estimation. To address these issues, we developed a modified LD-pruning algorithm that incorporates adjusted R^2 and checks for LD-matrix singularity to regulate variant selection. The modified algorithm follows the same selection-exclusion procedure as the standard version but retains a variant only if it increases the adjusted R^2 and does not make the LD matrix singular. Adjusted R^2 penalizes the inclusion of

additional variants and increases only when an added variant improves exposure prediction beyond chance (see the next section for details of the calculation). The advantage of the modified approach was demonstrated in the analysis of the *HP* gene region (see analysis using HP levels and variants in the *HP* gene region). Given its advantages, all subsequent comparisons with COJO, SuSiE, and PCA in both simulation analysis and across 15 gene regions were performed against the modified LD-pruning method. For both standard and modified LD-pruning, we explored a range of r^2 pruning thresholds from 0 to 0.8 in increments of 0.02.

COJO algorithm

Variant selection in COJO is based on conditional *p* values, in contrast to LD-pruning, which relies on marginal *p* values. The algorithm begins by selecting the variant with the smallest marginal *p* value below a pre-specified cutoff. For the remaining variants, the algorithm calculates their conditional *p* values, conditioned on the variant(s) already selected. It also checks for collinearity between the variant being tested and the selected variant(s), which is assessed by regressing the variant under consideration on the selected variants and calculating the proportion of variance explained (R^2). If R^2 exceeds a pre-specified collinearity threshold, the conditional *p* value for that variant is set to 1, effectively excluding it from selection. In comparison to pruning, this approach can detect not only high pairwise correlation between variants but also more complex patterns of collinearity (for example, where one variant's associations with the exposure can be expressed as a linear combination of other variants' associations). The variant with the smallest conditional *p* value (below the cutoff) is then added to the set of selected variants. The algorithm re-computes conditional *p* values for all selected variants in the model and then (if any variants have *p* values above the cutoff) removes the variant with the largest conditional *p* value. These steps are repeated until no variants are added to or removed from the model. The COJO algorithm outputs a final list of variants, where each variant has a conditional *p* value below the cutoff and a correlation with other selected variants below the collinearity threshold. The calculation of conditional *p* values using summary statistics is detailed in the original publications of the COJO algorithm.[7] For our analysis, we set the *p* value cutoff to 0.001 and explored a range of collinearity thresholds from 0.1 to 0.9 in increments of 0.1.

SuSiE algorithm

In the SuSiE framework, the vector of regression coefficients for associations between genetic variants and the exposure is modeled as the sum of a pre-specified number of single-effect vectors, each containing one non-zero element corresponding to a candidate causal variant.[8,9] Given K single-effect vectors, the algorithm iteratively updates these vectors in a cyclic manner (1,2, …, K and then back to $k = 1$). At each iteration, a single-effect vector is fitted based on the data and the current estimates of the other $K - 1$ single-effect vectors. This process continues until convergence is achieved. SuSiE outputs a posterior inclusion probability (PIP) for each variant, representing the probability that the variant is causal. Credible sets are then constructed based on the posterior distributions of the K single-effect vectors and a given purity (minimum absolute correlation across the credible set variants) of each set.[8] To form $100 \times \alpha$ % credible sets for a specific single-effect vector, variants are ranked by their single-effect-vector-specific PIPs in descending order and then sequentially added until the cumulative single-effect-vector PIP exceeds α.[8] For our analyses, we specified the number of single-effect vectors as 10 and constructed 95% credible sets ($\alpha = 0.95$), with purity set to 0.5. To generate a list of variants for IVs, we selected one variant to represent each credible set. Specifically, the variant with the largest PIP within each credible set was selected. In cases where multiple variants in the same credible set shared the highest PIP, one variant was randomly chosen.

PCA algorithm

In contrast to the other methods, the PCA algorithm[5] does not involve selecting specific genetic variants. Instead, it transforms the genetic variants into a lower-dimensional, orthogonal representation while preserving as much variance in the LD matrix as possible. These transformed variables are then used as IVs in the MR analysis. The algorithm begins with an eigendecomposition of a weighted version of the LD matrix, where the correlation at entry ij is weighted by $\frac{\widehat{\beta}_i}{SE_i} \times \frac{\widehat{\beta}_j}{SE_j}$ to prioritize variants with stronger variant-exposure associations. Here, $\widehat{\beta}_i$ and $\widehat{\beta}_j$ are the estimated associations of variants i and j with the exposure, and SE_i and SE_j are their corresponding standard errors. This step identifies eigenvalues and their corresponding eigenvectors, known as PCs. The top k PCs are then retained such that they explain a pre-specified threshold for the proportion of variance in the weighted LD matrix to be preserved. Subsequently, summary statistics of variant-exposure associations and the LD matrix are re-expressed using the selected PCs as the new basis. For our analysis, we explored a range of thresholds for the proportion of variance in the LD matrix explained by PCs, ranging from 90% to 99% in 1% increments and from 99.0% to 99.9% in 0.1% increments. Although PCA-based instruments are less interpretable at the variant level, in the context of MR, our primary interest is in the causal effect of the exposure on the outcome rather than the interpretability of individual instruments. Provided the resulting PCs satisfy the core IV assumptions, it is not critical whether instruments are constructed from PCs or from individual variants.

R^2

We used the proportion of variance explained, R^2, to measure instrument strength, as it is a direct indicator of statistical power,[12,13] with higher R^2 generally conferring greater power[12,13] in large-sample settings where weak instrument bias is negligible (see the discussion section below). R^2 was calculated from the F-statistic. Let N be the exposure GWAS sample size and J the number of IVs. Let $\widehat{\gamma} \in R^J$ be the vector of multivariable variant-exposure association estimates (from regression of the exposure on variants jointly) and let $\widehat{\Sigma_\gamma} \in R^{J \times J}$ be their covariance matrix. Then,

$$\widehat{F} = \frac{N - J}{J} \widehat{\gamma}^T \widehat{\Sigma_\gamma^{-1}} \widehat{\gamma}.$$

We construct $\widehat{\gamma}$ and $\widehat{\Sigma_\gamma}$ from univariable variant-exposure association estimates, their standard errors, and the LD matrix (between variants). Full derivations of $\widehat{\gamma}$ and $\widehat{\Sigma_\gamma}$ are provided in the supplemental information. The F-statistic formula for correlated variants presented here builds on existing results in the literature but, to our knowledge, has not been published previously. For the PCA approach, we first transform the univariable variant-exposure association estimates and the LD matrix to the basis defined by the retained PCs in the analysis (as described earlier). The resulting transformed quantities are then used to construct $\widehat{\gamma}$ and $\widehat{\Sigma_\gamma}$ and to compute $\widehat{F}$ as above, except that now these are multivariable genetic associations with the PCs, not with single variants. Finally, R^2 is obtained from the F-statistic via

$$\widehat{R^2} = \frac{J\,\widehat{F}}{N - J - 1 + J\,\widehat{F}}.$$

In the modified LD-pruning method, adjusted R^2 (R^2_{adj}) is used to regulate variant selection, retaining only variants that can increase R^2_{adj}. It is calculated from R^2 as

$$\widehat{R^2_{adj}} = 1 - \frac{\left(1 - \widehat{R^2}\right)(N - 1)}{N - J - 1},$$

where N is the exposure GWAS sample size and J is the number of IVs.

Analysis using HP levels and variants in the *HP* gene region

Using circulating HP levels and variants in the *HP* gene region as a motivating example, we first illustrated the advantages of the modified LD-pruning method over the standard approach and then evaluated whether variant sets derived from the modified LD-pruning, COJO, SuSiE, and PCA methods could offer a notable gain in instrument strength compared to the lead-variant-only approach. Improvements in instrument strength were assessed by examining the variance explained and the precision of MR estimates. For this analysis, we included 1,620 variants in the *cis*-region (±500 kb of the protein-coding region) of the *HP* gene. The calculation of variance explained requires summary statistics for variant-protein association estimates, their standard errors, and the LD matrix. We obtained summary statistics for variant-HP association estimates and their standard errors from the Fenland study,[14] which included 10,708 participants of European ancestry. The LD matrix was estimated using data from 356,557 unrelated European-ancestry participants in the UK Biobank.[15] For the MR analyses, we used red blood cell count (RBC) as the outcome, examining the association of genetically predicted HP with RBC. HP is an acute-phase glycoprotein predominantly synthesized and secreted by hepatocytes.[16] Its main function is to scavenge free hemoglobin released from erythrocytes with high affinity, thereby preventing hemoglobin-mediated oxidative damage.[16] To the best of our knowledge, genetic links between HP and RBC have not previously been examined. We note that this analysis was conducted primarily for illustrative purposes, with an emphasis on comparing the precision of MR estimates rather than interpreting the causal estimates. Summary statistics for variant-RBC association were obtained from the UK Biobank using linear regression, adjusted for age, sex, assessment center, and the top 40 genetic PCs provided by the UK Biobank[15] to control for population stratification. Please note that these covariate PCs are distinct from the PCs used in the PCA approach. MR estimates were computed using the inverse-variance weighted method with a fixed-effects model, accounting for LD between variants (or PCs). When selected variants are highly correlated, the LD matrix may become singular (non-invertible), making MR estimation impossible. In such cases, we stabilized the inversion of the LD matrix by adding a small constant $\varepsilon = -\lambda_{min} + 10^{-8}$ to its diagonal, where λ_{min} is the smallest eigenvalue of the original LD matrix. This adjustment ensures all eigenvalues are positive and guarantees the invertibility of the LD matrix. LD matrices were computed using PLINK v.1.9. MR analyses were performed using the MendelianRandomization package (v.0.10.0) in R (v.4.3.1).

Simulation analysis

In the HP example, since the true underlying genetic variance explained is unknown, it is difficult to assess the extent to which the observed gain in variance explained truly reflects the variance explained by IVs. Some of the apparent gain in precision may be spurious, potentially inflated by mis-estimation of the correlations between variants. Such mis-estimation can arise for many reasons: estimates may be obtained in a mismatched population, or they may simply be estimated with uncertainty. When the variant correlation matrix is ill-conditioned, even imperceptible differences in the matrix can lead to large changes in MR estimates.[5] To complement the findings from the HP analysis, we conducted a simulation study in which the true genetic variance of the simulated traits is known, allowing us to evaluate how reliably variants derived from each method capture the true genetic contribution.

Our simulation design was informed by the HP example. Individual-level genetic data for 1,620 variants in the *cis*-region of the *HP* gene were obtained from the UK Biobank, on 20,000 randomly selected unrelated individuals with complete genotyping data. We considered two scenarios: one with a single causal variant and another with two causal variants. In both scenarios, the causal variants were set to explain 40% of the trait variance. To simplify the data-generating process, genotyping data were standardized. For the single-causal-variant scenario, one variant was randomly selected as the causal variant. Its effect size was set to $\sqrt{0.4}$, ensuring that the causal variant explained 40% of the variance in the trait. For the two-causal-variants scenario, one variant was randomly chosen to serve as the first causal variant. A second variant was then selected such that its covariance with the first causal variant was approximately 0.5, ensuring moderate correlation between the two causal variants. The effect sizes for the two causal variants were set to $\sqrt{\frac{0.4}{3}}$, ensuring that each causal variant individually explained 30% and the two causal variants collectively explained 40% of the variance in the trait. Each simulation experiment was repeated 100 times for both scenarios. At each repetition, we calculated genetic association estimates for each variant in turn. These summarized data and an estimate of the LD matrix (for modified LD-pruning, SuSiE, and PCA) or individual-level genotype data (for COJO) were used by the variant selection methods.

The data-generating model for traits with one causal variant is

$$X_i = \sqrt{0.4}\,G_i + \varepsilon_i,$$

where X_i is the simulated trait for individual I, G_i is the genotyping data for the causal variant, and $\varepsilon_i \sim N(0, 0.6)$ is the residual error term with a variance of 60%.

The data-generating model for traits with two causal variants is

$$X_i = \sqrt{\frac{0.4}{3}}G_{i1} + \sqrt{\frac{0.4}{3}}G_{i2} + \varepsilon_i,$$

where X_i is the simulated trait for individual I, G_{i1} is the genotyping data for the first causal variant, G_{i2} is the genotyping data for the second causal variant, and $\varepsilon_i \sim N(0, 0.6)$ is the residual error term with a variance of 60%.

To further evaluate the performance of the four variant selection strategies under more realistic conditions, we conducted two extended simulation studies. In extended simulation study I, the true causal variant and its proxies were removed

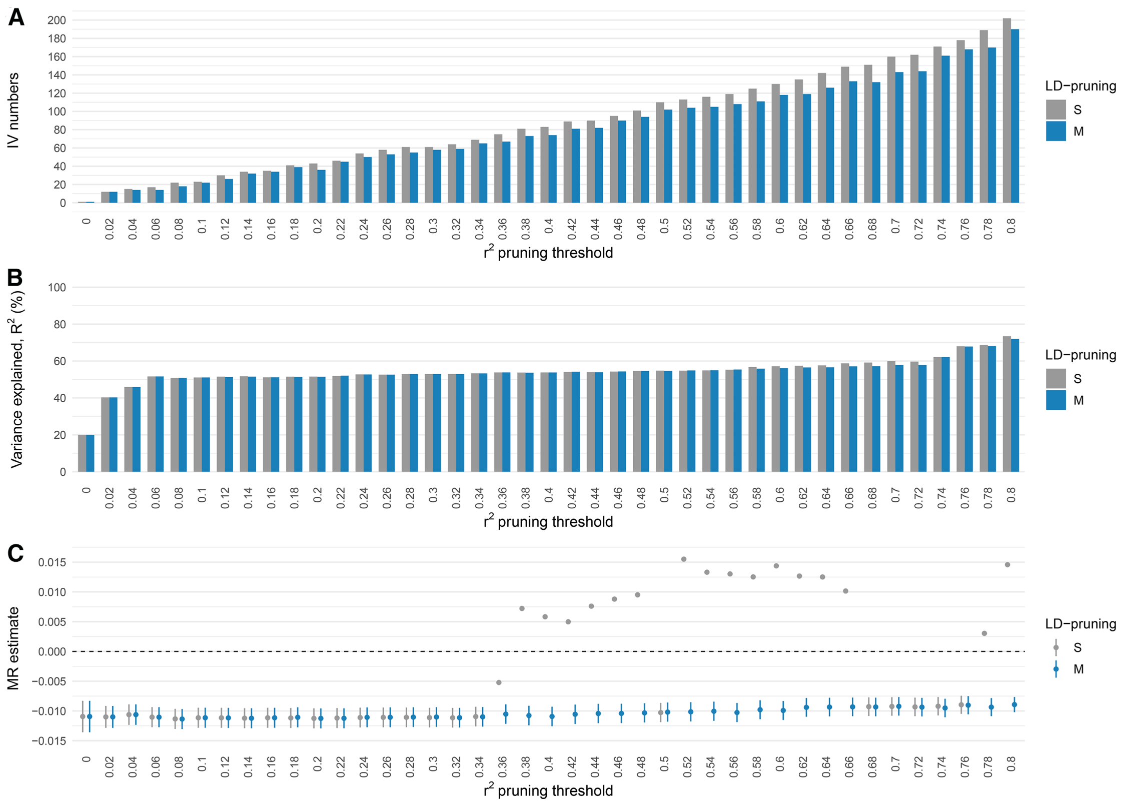

Figure 1. Comparison of standard and our proposed modified LD-pruning methods for variant selection in the *Haptoglobin* gene region
(A) Number of variants retained as IVs.
(B) Proportion of variance in haptoglobin levels explained.
(C) MR estimate for the effect of haptoglobin on red blood cell count.
S, standard LD-pruning method; M, modified LD-pruning method. Error bars represent 95% confidence intervals (CIs).

from the dataset, simulating the common practical situation in which the true underlying causal variants are unknown and may not be directly genotyped. Extended simulation study II focused on horizontal pleiotropy and considered a scenario in which two independent causal variants influence the exposure, with one of these variants also exerting a direct (pleiotropic) effect on the outcome. Detailed descriptions of these two studies are provided in the supplemental information.

Analysis across 15 gene regions

To assess the generalizability of our findings from the *HP* gene region to other regions, we extended our analysis to 15 additional gene regions, each characterized by varying proportions of *cis*-genetic variance contributed by non-lead *cis*-protein quantitative trait loci (pQTLs).[14] For this analysis, summary statistics for variant-protein association estimates and their standard errors were obtained from the Fenland study.[14] The LD matrices for each gene region were estimated using data from 356,557 unrelated European-ancestry participants in the UK Biobank. The selected 15 gene regions are scattered across 10 chromosomes and contain 1 to 9 *cis*-pQTLs that explain 0.5%–49.1% of the variance in their corresponding proteins, with the relative proportion of variance explained by non-lead *cis*-pQTLs as previously estimated ranging from 0% to 70%. Summary information for the 15 gene regions is provided in Table S3.

Results

Figure 1 compares standard LD-pruning with our proposed modified LD-pruning method, which adds adjusted R^2 and LD-matrix singularity checks to regulate variant selection, in the *HP* gene region. At a given r^2 threshold, the standard method generally retained slightly more variants than the modified version (Figure 1A), but both explained a similar proportion of variance (Figure 1B). MR estimates from the modified method were stable across r^2 thresholds, whereas the standard method could produce anomalous estimates with reversed direction and implausibly small standard errors (Figure 1C), even at moderate thresholds ($r^2 < 0.4$). These anomalies are likely caused by multicollinearity among the selected variants. Based on these findings, all subsequent analyses were conducted using our proposed modified LD-pruning method unless specified otherwise.

Figure 2 summarizes the results for variance explained by variant sets selected using the four methods, with results for modified LD-pruning, COJO, and PCA shown across different tuning parameters. IVs incorporating non-lead variants explained more variance than the lead variant alone ($R^2 = 20\%$; Figure 2). For the modified LD-pruning method, increasing the pruning threshold

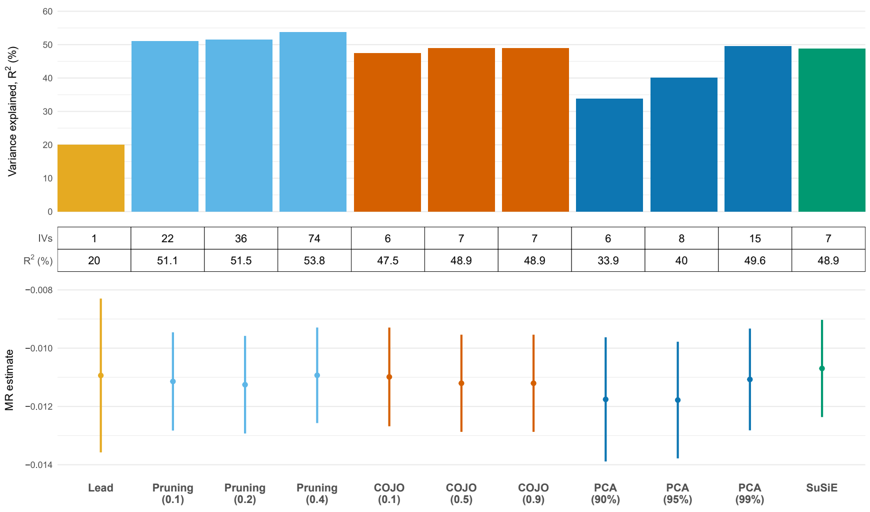

Figure 2. Proportion of variance in haptoglobin levels explained by variants selected using modified LD-pruning, COJO, SuSiE, and PCA, together with the corresponding MR estimates for the effect on red blood cell count
The table below the plot shows the number of IVs and variance explained, R^2 (%). Error bars represent 95% CIs. For modified LD-pruning, results are shown for pruning thresholds of $r^2 = 0.1$, 0.2, and 0.4. For PCA, results correspond to PCs capturing 90%, 95%, and 99% of the variance in the LD matrix. For COJO, results are shown for collinearity thresholds of 0.1, 0.5, and 0.9.

substantially increased the number of variants retained as IVs (from 22 at $r^2 = 0.1$ to 74 at $r^2 = 0.4$), yet R^2 rose only modestly, from 51.1% to 53.8% (Figure 2). Variant selection using COJO was little affected by the collinearity threshold; across thresholds of 0.1, 0.5, and 0.9, it consistently selected 6–7 variants and yielded stable R^2 values ranging from 47.5% to 48.9% (Figure 2). PCA using 6, 8, and 15 PCs (explaining 90%, 95%, and 99% of the LD matrix, respectively) yielded R^2 values of 33.9%, 40.0%, and 49.6%. For SuSiE, selecting the variant with the highest PIP per credible set explained an R^2 of 48.9%. We also evaluated R^2 across alternative combinations of variants, selecting one variant from each credible set irrespective of its PIP; in each case, the R^2 was within 0.2 percentage points of the value based on the highest-PIP variant from each credible set. Given these negligible differences, we maintain the strategy of selecting the highest PIP variant from each credible set for the SuSiE method for the remainder of the manuscript. Further, improvements in R^2 from variant selection methods that incorporate non-lead variants translate into notable gains in the precision of MR estimates, as evidenced by narrower 95% confidence intervals (Figure 2). Full results across all tuning parameters are provided in Table S1.

Figure 3 shows results from simulation analyses with one and two true causal variants. In both settings, modified LD-pruning, COJO, and PCA at higher tuning parameters, as well as SuSiE, yielded median R^2 values that closely matched the expected value of 40% (Figure 3), although results from modified LD-pruning at the $r^2 < 0.4$ threshold were slightly optimistic, with the approach finding variants that explained 40.2% and 40.3% of the variance in the one- and two-causal-variant scenarios, respectively. It is worth noting that in the two-causal-variants scenario (Figure 3B), the lead variant alone explained only 30.4% of the variance, as expected. Full results across all tuning parameters are provided in Table S2. Further, in extended simulation study I, when the true causal variant and its proxies (at three correlation thresholds of $r^2 > 0.95$, 0.8, and 0.4) were removed, all four strategies that incorporate non-lead variants recovered more genetic variance than the lead-variant-only approach (Figure S2), most likely because these additional non-lead variants partially capture information about the removed causal variant. Notably, this disparity became more pronounced as more proxies were removed (i.e., as the correlation threshold was lowered from $r^2 > 0.95$ to $r^2 > 0.8$ and then to $r^2 > 0.4$; Figure S2), further weakening the performance of the lead-variant-only approach. In extended simulation study II, where horizontal pleiotropy was introduced, MR estimates from all four strategies exhibited bias, presumably due to inclusion of the pleiotropic variant or variants in LD with it (Figure S3).

Figure 4 displays the variance explained by each method across 15 gene regions, with full results across all tuning parameters provided in Table S4. To facilitate direct comparison with the lead-variant-only approach, we calculated the normalized R^2, defined as each method's R^2 divided by the R^2 from the lead variant alone. Figure 5 summarizes the distribution of normalized R^2 values for the four variant selection methods across 15 gene regions. Across all regions, the median normalized R^2 exceeded 1 (ranging from 1.80 to 4.95), and variability between methods appeared to be greater in regions with higher median normalized R^2. Figure 6 shows the distribution of method rankings based on normalized R^2 across the 15 gene regions. Rankings were generally stable across regions, with SuSiE showing the greatest variability. Within each method, higher tuning parameters tended to yield higher normalized R^2 and better ranks. Across methods, modified LD-pruning consistently ranked highest, followed by COJO. PCA at 99% generally outperformed SuSiE, with PCA at 90% consistently ranked lowest.

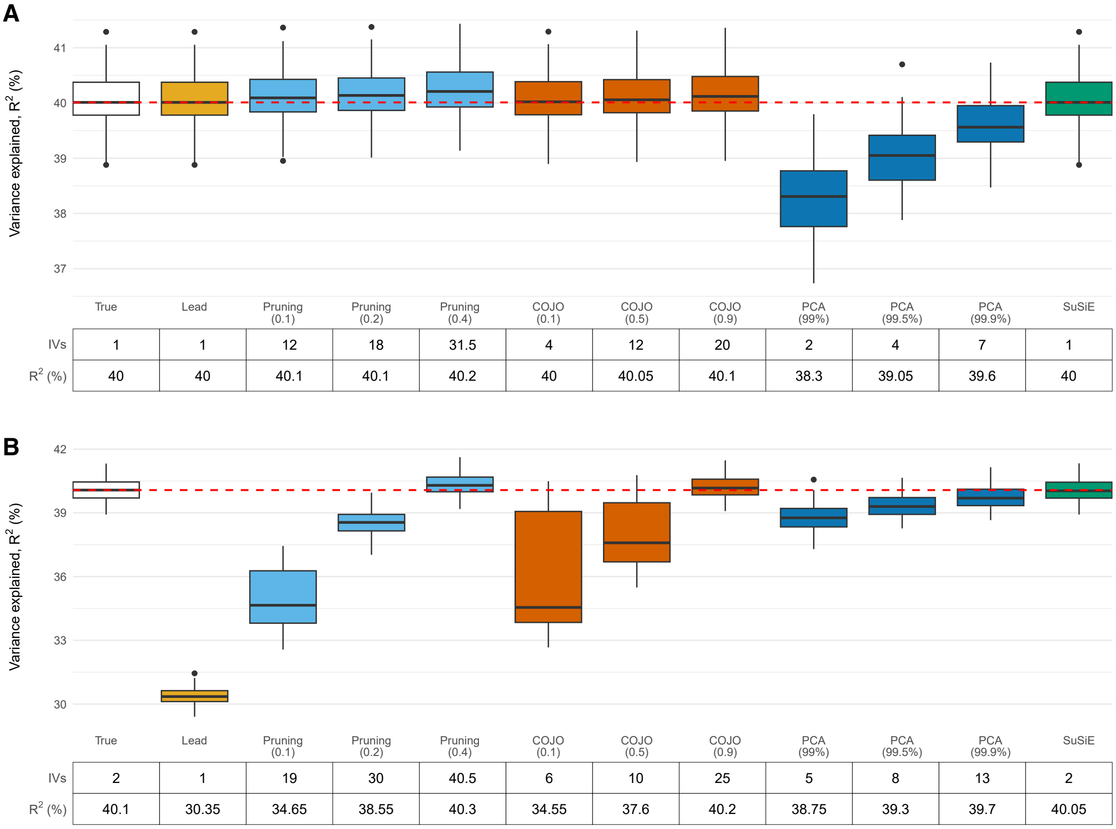

	True	Lead	Pruning (0.1)	Pruning (0.2)	Pruning (0.4)	COJO (0.1)	COJO (0.5)	COJO (0.9)	PCA (99%)	PCA (99.5%)	PCA (99.9%)	SuSiE
IVs	1	1	12	18	31.5	4	12	20	2	4	7	1
R^2 (%)	40	40	40.1	40.1	40.2	40	40.05	40.1	38.3	39.05	39.6	40

	True	Lead	Pruning (0.1)	Pruning (0.2)	Pruning (0.4)	COJO (0.1)	COJO (0.5)	COJO (0.9)	PCA (99%)	PCA (99.5%)	PCA (99.9%)	SuSiE
IVs	2	1	19	30	40.5	6	10	25	5	8	13	2
R^2 (%)	40.1	30.35	34.65	38.55	40.3	34.55	37.6	40.2	38.75	39.3	39.7	40.05

Figure 3. Proportion of variance in simulated traits with one or two causal variants explained by variants selected using modified LD pruning, COJO, SuSiE, and PCA

(A) One causal variant.

(B) Two causal variants.

Each simulation was repeated 100 times. The table below each plot shows the median number of IVs and median R^2 (%) across 100 replicates. The red dashed line indicates the median true R^2 (%), calculated from regressing the exposure on the true causal variant(s) using individual-level data. Boxplots represent the estimates from each replicate. The box displays the lower quartile, median, and upper quartile; whiskers extend to the minimum and maximum values within 1.5× the interquartile range from the lower and upper quartiles. Estimates outside this range are shown as individual points. For modified LD-pruning, results are shown for pruning thresholds of $r^2 = 0.1$, 0.2, and 0.4. For PCA, results correspond to PCs capturing 99%, 99.5%, and 99.9% of variance in the LD matrix. For COJO, results are shown for collinearity thresholds of 0.1, 0.5, and 0.9.

Discussion

Instrument selection is crucial in MR. The ideal variant set in a *cis*-MR analysis would consist of all variants in a gene region that independently contribute to the variance of the trait. However, selecting all such variants is rarely feasible in practice due to LD. In this study, we evaluated the predictive strength of variant sets obtained using four variant selection methods as measured by the proportion of variance in the exposure explained. We first assessed their predictive strength using HP as a motivating example. We then extended the evaluation to simulated traits with known variance explained by the causal variants and finally to fifteen proteins, each exhibiting varying contributions of *cis*-genetic variance from non-lead *cis*-pQTLs.

Is there value in incorporating correlated non-lead variants as IVs in *cis*-MR analyses?

Several lines of evidence from our analysis support the added value of including non-lead variants as IVs in *cis*-MR analysis. Firstly, gains in variance explained from incorporating non-lead variants were consistently observed across all four variant selection methods and across different gene regions. Secondly, as seen in the HP example, the inclusion of non-lead variants can notably improve the precision of MR estimates. Third, simulation analyses with two causal variants demonstrated that the lead variant alone can fail to capture the full genetic variance, highlighting the potential benefit of including additional non-lead variants, even if moderately correlated with the lead variant. Further, as illustrated in the simulation scenario where the true causal variant was not present in the dataset, methods incorporating non-lead variants allowed recovery of more genetic variance than the lead-variant-only approach. This advantage became increasingly apparent as more proxies of the causal variant were removed from the dataset and the lead variant alone became less informative about the removed causal variant. These findings again support the potential benefit of including additional non-lead variants as IVs.

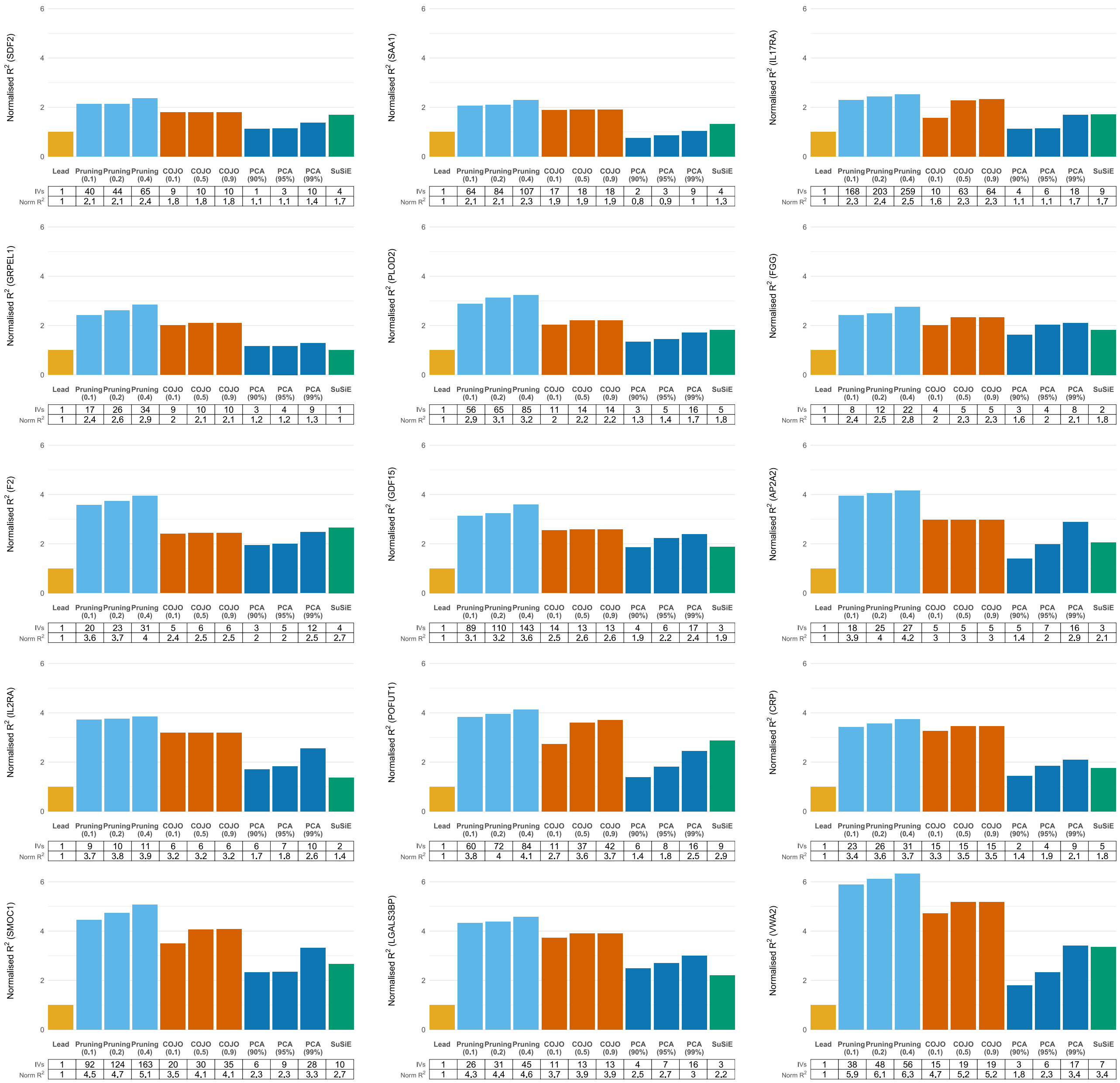

Figure 4. Proportion of variance in protein levels explained by variants selected using modified LD pruning, COJO, SuSiE, and PCA across 15 gene regions

Normalized R^2 is calculated as each method's R^2 divided by the R^2 from the lead variant alone. The table below each plot shows the number of IVs and the corresponding normalized R^2. For modified LD-pruning, results are shown for pruning thresholds of $r^2 = 0.1$, 0.2, and 0.4. For PCA, results correspond to PCs capturing 90%, 95%, and 99% of variance in the LD matrix. For COJO, results are shown for collinearity thresholds of 0.1, 0.5, and 0.9.

Together, findings from our empirical and simulated data suggest that incorporating non-lead variants can potentially boost the statistical power of *cis*-MR analyses.

Which method performs best?

Given that our analyses provide strong support for incorporating non-lead variants in variant selection, a natural question is which method performs best. We acknowledge that the comparison between methods is not fully fair. For instance, PCA with a higher threshold that results in the inclusion of more PCs will always lead to a higher R^2 statistic. Our priority in this manuscript is to compare methods as they are likely to be implemented in practice. All methods are compared based on access to the same data, so the comparison is equitable. Across the *HP* gene region and 15 regions examined, modified LD-pruning appeared to perform best in terms of variance explained, with pruning at an r^2 of 0.4 consistently ranking highest. However, it is also important to note that our simulation analyses have revealed a slight upward bias in R^2 estimates for the modified LD-pruning method as the pruning threshold increases (Table S2), suggesting that R^2 may

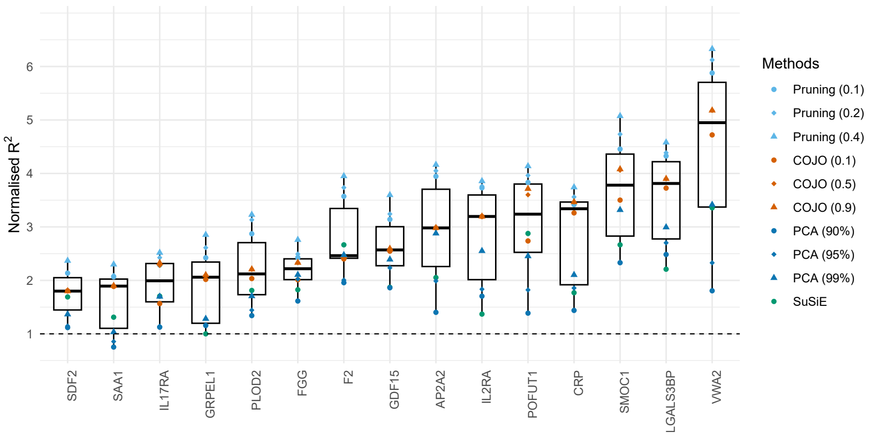

Figure 5. Distribution of normalized variance explained by variants selected using modified LD-pruning, COJO, SuSiE, and PCA across 15 gene regions
Normalized R^2 is calculated as each method's R^2 divided by the R^2 from the lead variant alone. For modified LD-pruning, results are shown for pruning thresholds of $r^2 = 0.1$, 0.2, and 0.4. For PCA, results correspond to PCs capturing 90%, 95%, and 99% of variance in the LD matrix. For COJO, results are shown for collinearity thresholds of 0.1, 0.5, and 0.9. The box displays the lower quartile, median, and upper quartile; whiskers extend to the minimum and maximum values within 1.5× the interquartile range from the lower and upper quartiles.

have been modestly overestimated at moderate r^2 thresholds (e.g., $r^2 = 0.4$) in practice.

We argue that selecting a single "best" method may be unnecessary. Instead, observing similar results from multiple variant selection approaches can enhance the robustness of *cis*-MR findings. While incorporating non-lead variants as IVs can improve instrument strength, it is important to acknowledge that non-lead variants could potentially make the variant set more prone to horizontal pleiotropy or confounding by LD, leading to biased MR estimates. Horizontal pleiotropy occurs when a genetic variant influences the outcome independently of the exposure of interest, violating a key assumption of MR and invalidating its causal inference.[2] In the context of *cis*-MR, horizontal pleiotropy could arise if non-lead variants in the *cis*-region of the protein of interest (protein A) are also *trans*-pQTLs for another protein (protein B), which directly affects the outcome independently of protein A. Confounding by LD would arise if selected variants in the *cis*-region of protein A are correlated with variants affecting the outcome via another causal pathway, not via protein A. Including variants that either affect another pathway or are correlated with a variant affecting another pathway can introduce bias in estimating the causal effect of the protein of interest on the outcome. For example, the *ABO* locus is known to be highly pleiotropic, with six independent variants at the locus reported as *trans*-pQTLs affecting 14 different plasma proteins.[17] Given the potential for pleiotropy, using multiple variant selection approaches can be advantageous. Consistency in MR estimates across variant sets with differing sets of non-lead variants can strengthen confidence that the observed findings reflect a true causal relationship. However, as demonstrated in extended simulation study II, in unfavorable scenarios involving horizontal pleiotropy, all variant selection strategies incorporating non-lead variants can also fail simultaneously. Therefore, agreement across methods should not be interpreted as definitive evidence that pleiotropy is absent.

Recommendations

Balancing the benefits of increased power from incorporating non-lead variants with the potential risks of pleiotropy and overprecision in estimates, we recommend that researchers using variant selection strategies that incorporate non-lead variants additionally report results using

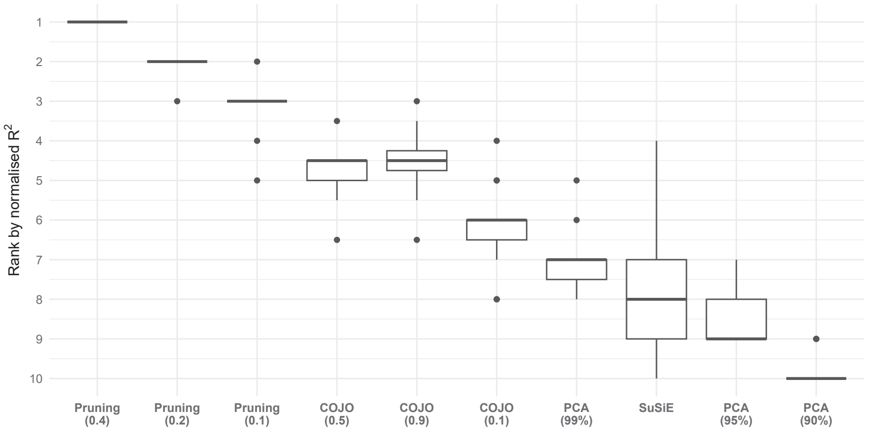

Figure 6. Distribution of method rankings based on normalized variance explained across 15 gene regions
Each method's rank reflects its relative performance within a gene region, with rank 1 indicating the highest normalized R^2. Normalized R^2 is calculated as each method's R^2 divided by the R^2 from the lead variant alone. For modified LD-pruning, results are shown for pruning thresholds of $r^2 = 0.1$, 0.2, and 0.4. For PCA, results correspond to PCs capturing 90%, 95%, and 99% of variance in the genetic correlation matrix. For COJO, results are shown for collinearity thresholds of 0.1, 0.5, and 0.9. The box displays the lower quartile, median, and upper quartile; whiskers extend to the minimum and maximum values within 1.5× the interquartile range from the lower and upper quartiles. Ranks outside this range are shown as individual points.

the lead-variant-only approach. If the MR estimate based on multiple variants is slightly or moderately more precise than the estimate based on the lead variant only, then it is plausibly reliable. If it is much more precise or the point estimate differs sharply, then numerical instability may be suspected. To ensure robustness of findings to the choice of non-lead variants, we also recommend investigating IV sets derived from different selection methods (such as those evaluated in this study). Consistent MR estimates across IVs with varying compositions of non-lead variants strengthen confidence in causal inferences. Additionally, where feasible, the analysis plan should include strategies to detect and mitigate potential pleiotropy. While we noted earlier that incorporating non-lead variants may potentially increase the risk of pleiotropy, this does not imply that the lead variant is immune. Indeed, any variant, including the lead one, could potentially exert pleiotropic effects. Positive and negative control analyses can help flag potential pleiotropic effects. If pleiotropy is suspected based on prior literature or secondary/sensitivity analyses and the pleiotropic pathway is known, multivariable MR can be employed to adjust for pleiotropic effects and enhance the reliability of causal inferences.[18] Further, a recently proposed conditional MR framework,[19] which integrates conditional analysis into the standard MR framework by modeling conditional variant-exposure and variant-outcome associations, can be used to reduce LD-induced pleiotropy. Additionally, colocalization can help distinguish causation from confounding by LD.[20]

Beyond variance explained

There are many factors to consider in variant selection. Our investigation is limited to statistical aspects and does not consider the biological context of molecular traits used to guide the variant selection process. Molecular traits, such as protein levels and gene expression, can vary substantially across tissues and under different physiological or pathological conditions. In practice, it is often challenging to pinpoint the mechanism-relevant tissues for a given outcome, let alone obtain molecular trait data from relevant tissues under the appropriate physiological or pathological conditions. If the molecular trait data used to guide variant selection are biologically irrelevant, then including non-lead variants even if they increase variance explained may not meaningfully improve the power of the MR analysis. In such cases, it may be preferable to guide variant selection using phenotypes that lie downstream in the biological pathway through which the molecular trait exerts its effect.[21] It would still be possible to use the methods evaluated here to select non-lead variants for downstream phenotypes, but secondary signals are less commonly observed for these traits than for molecular traits, as genetic variants typically explain less variance in traits that lie further downstream in the biological pathway from the molecular trait.

In our study, we used the proportion of variance explained, R^2, to measure instrument strength, as our focus is on statistical power, to which R^2 is directly linked,[12,13] in contrast to alternatives such as adjusted R^2 or F-statistic. While the F-statistic is valuable for informing weak instrument bias,[1] our study assumes a large sample size and a two-sample setting, where such bias is generally not a concern[17] (and, even if present, would bias associations toward the null and would not lead to inflated type 1 error rates and false-positive findings). However, if the investigation is performed in the one-sample setting with a relatively small sample size, weak instrument bias could arise and bias associations toward the confounded observational estimate.[17] In such cases, assessing the performance of IVs solely by variance explained may not be sufficient. Investigators are recommended to also report and monitor the F-statistic (e.g., ensuring F-statistic > 10) to balance gains in power against potential bias. Finally, we note that the R^2 values reported in our study are in-sample estimates, obtained using the same data to both select and evaluate the IVs, and may therefore be mildly more optimistic than the true R^2. However, because we use R^2 only to compare the relative performance of different variant selection methods, any optimism in the in-sample R^2 is unlikely to materially affect the ranking of methods.

To summarize, our findings, supported by empirical and simulated data, suggest that variant selection strategies incorporating correlated non-lead variants into a gene region can reliably increase instrument strength for *cis*-MR analyses compared to the lead variant alone. We recommend adopting such strategies but advise against checking estimates against the estimate using the lead variant only to detect potential numerical instability.

Data and code availability

All summary statistics for variant-protein association estimates used in this study were downloaded from https://omicscience.org/apps/covidpgwas/. Analysis code is available at https://github.com/az452/cis-MR-variant-selection.git.

Web resources

Online Mendelian Inheritance in Man (OMIM), http://www.omim.org

Acknowledgments

S.B., A.Z., J.P., and V.K. are supported by the Wellcome Trust (225790/Z/22/Z) and the United Kingdom Research and Innovation Medical Research Council (MC_UU_00002/7).

Declaration of interests

The authors declare no competing interests.

Supplemental information

Supplemental information can be found online at https://doi.org/10.1016/j.xhgg.2026.100573.

Received: September 10, 2025
Accepted: January 9, 2026

References

1. Burgess, S., Thompson, S.G.; and CRP CHD Genetics Collaboration (2011). Avoiding bias from weak instruments in Mendelian randomization studies. Int. J. Epidemiol. *40*, 755–764. https://doi.org/10.1093/ije/dyr036.
2. Burgess, S., Davey Smith, G., Davies, N.M., Dudbridge, F., Gill, D., Glymour, M.M., Hartwig, F.P., Kutalik, Z., Holmes, M.V., Minelli, C., et al. (2019). Guidelines for performing Mendelian randomization investigations: update for summer 2023. Wellcome Open Res. *4*, 186. https://doi.org/10.12688/wellcomeopenres.15555.3.
3. Gill, D., Georgakis, M.K., Walker, V.M., Schmidt, A.F., Gkatzionis, A., Freitag, D.F., Finan, C., Hingorani, A.D., Howson, J.M.M., Burgess, S., et al. (2021). Mendelian randomization for studying the effects of perturbing drug targets. Wellcome Open Res. *6*, 16. https://doi.org/10.12688/wellcomeopenres.16544.2.
4. Sun, B.B., Chiou, J., Traylor, M., Benner, C., Hsu, Y.-H., Richardson, T.G., Surendran, P., Mahajan, A., Robins, C., Vasquez-Grinnell, S.G., et al. (2023). Plasma proteomic associations with genetics and health in the UK Biobank. Nature *622*, 329–338. https://doi.org/10.1038/s41586-023-06592-6.
5. Burgess, S., Zuber, V., Valdes-Marquez, E., Sun, B.B., and Hopewell, J.C. (2017). Mendelian randomization with fine-mapped genetic data: Choosing from large numbers of correlated instrumental variables. Genet. Epidemiol. *41*, 714–725. https://doi.org/10.1002/gepi.22077.
6. Hutchinson, A., Asimit, J., and Wallace, C. (2020). Fine-mapping genetic associations. Hum. Mol. Genet. *29*, R81–R88. https://doi.org/10.1093/hmg/ddaa148.
7. Yang, J., Ferreira, T., Morris, A.P., Medland, S.E., Genetic Investigation of ANthropometric Traits GIANT Consortium, DIAbetes Genetics Replication And Meta-analysis DIAGRAM Consortium, Madden, P.A.F., Heath, A.C., Martin, N.G., Montgomery, G.W., et al. (2012). Conditional and joint multiple-SNP analysis of GWAS summary statistics identifies additional variants influencing complex traits. Nat. Genet. *44*, 369–375. S1–S3. https://doi.org/10.1038/ng.2213.
8. Wang, G., Sarkar, A., Carbonetto, P., and Stephens, M. (2020). A Simple New Approach to Variable Selection in Regression, with Application to Genetic Fine Mapping. J. R. Stat. Soc. Ser. B Stat. Methodol. *82*, 1273–1300. https://doi.org/10.1111/rssb.12388.
9. Zou, Y., Carbonetto, P., Wang, G., and Stephens, M. (2022). Fine-mapping from summary data with the "Sum of Single Effects" model. PLoS Genet. *18*, e1010299. https://doi.org/10.1371/journal.pgen.1010299.
10. Benner, C., Spencer, C.C.A., Havulinna, A.S., Salomaa, V., Ripatti, S., and Pirinen, M. (2016). FINEMAP: efficient variable selection using summary data from genome-wide association studies. Bioinformatics *32*, 1493–1501. https://doi.org/10.1093/bioinformatics/btw018.
11. Karhunen, V., Launonen, I., Järvelin, M.-R., Sebert, S., and Sillanpää, M.J. (2023). Genetic fine-mapping from summary data using a nonlocal prior improves the detection of multiple causal variants. Bioinformatics *39*, btad396. https://doi.org/10.1093/bioinformatics/btad396.
12. Burgess, S. (2014). Sample size and power calculations in Mendelian randomization with a single instrumental variable and a binary outcome. Int. J. Epidemiol. *43*, 922–929. https://doi.org/10.1093/ije/dyu005.
13. Pierce, B.L., Ahsan, H., and VanderWeele, T.J. (2011). Power and instrument strength requirements for Mendelian randomization studies using multiple genetic variants. Int. J. Epidemiol. *40*, 740–752. https://doi.org/10.1093/ije/dyq151.
14. Pietzner, M., Wheeler, E., Carrasco-Zanini, J., Raffler, J., Kerrison, N.D., Oerton, E., Auyeung, V.P.W., Luan, J., Finan, C., Casas, J.P., et al. (2020). Genetic architecture of host proteins involved in SARS-CoV-2 infection. Nat. Commun. *11*, 6397. https://doi.org/10.1038/s41467-020-19996-z.
15. Bycroft, C., Freeman, C., Petkova, D., Band, G., Elliott, L.T., Sharp, K., Motyer, A., Vukcevic, D., Delaneau, O., O'Connell, J., et al. (2018). The UK Biobank resource with deep phenotyping and genomic data. Nature *562*, 203–209. https://doi.org/10.1038/s41586-018-0579-z.
16. di Masi, A., De Simone, G., Ciaccio, C., D'Orso, S., Coletta, M., and Ascenzi, P. (2020). Haptoglobin: From hemoglobin scavenging to human health. Mol. Aspect. Med. *73*, 100851. https://doi.org/10.1016/j.mam.2020.100851.
17. Suhre, K., Arnold, M., Bhagwat, A.M., Cotton, R.J., Engelke, R., Raffler, J., Sarwath, H., Thareja, G., Wahl, A., DeLisle, R.K., et al. (2017). Connecting genetic risk to disease end points through the human blood plasma proteome. Nat. Commun. *8*, 14357. https://doi.org/10.1038/ncomms14357.
18. Sanderson, E. (2021). Multivariable Mendelian Randomization and Mediation. Cold Spring Harb. Perspect. Med. *11*, a038984. https://doi.org/10.1101/cshperspect.a038984.
19. Lin, Z. (2024). A novel framework with automated horizontal pleiotropy adjustment in mendelian randomization. HGG Adv. *5*, 100339. https://doi.org/10.1016/j.xhgg.2024.100339.
20. Zuber, V., Grinberg, N.F., Gill, D., Manipur, I., Slob, E.A.W., Patel, A., Wallace, C., and Burgess, S. (2022). Combining evidence from Mendelian randomization and colocalization: Review and comparison of approaches. Am. J. Hum. Genet. *109*, 767–782. https://doi.org/10.1016/j.ajhg.2022.04.001.
21. Gill, D., Dib, M.-J., Cronjé, H.T., Karhunen, V., Woolf, B., Gagnon, E., Daghlas, I., Nyberg, M., Drakeman, D., and Burgess, S. (2024). Common pitfalls in drug target Mendelian randomization and how to avoid them. BMC Med. *22*, 473. https://doi.org/10.1186/s12916-024-03700-9.

HGGA, Volume *7*

Supplemental information

Variant selection to maximize variance explained in *cis*-Mendelian randomization

Ang Zhou, Ville Karhunen, Haodong Tian, Janne Pott, Ashish Patel, Eric A.W. Slob, and Stephen Burgess

1. Calculation of F-statistics and R^2 with correlated variants using GWAS summary statistics

A: Linear IV model with multiple (potentially correlated) IVs

Let Y denote an outcome, X a potential risk factor for the outcome, and $Z = (Z_1, \ldots, Z_J)'$ a J-vector of potentially correlated IVs. Then, we consider the linear IV model

$$Y = \alpha + X\theta_0 + U \quad (1)$$

$$X = \omega + Z'\gamma + V \quad (2)$$

where the error terms satisfy $E[U|Z] = 0$, $E[V|Z] = 0$. Since we are working with genetic association summary data, we are forced to make the assumption that these structural errors are homoskedastic, $E[U^2|Z] = \sigma_U^2$ and $E[V^2|Z] = \sigma_V^2$.

B: F-statistics under individual-level data

For a sample size N, an estimate of γ from the first-stage linear regression of X on Z is given by $\widehat{\gamma} = \widehat{var}(Z)^{-1}\widehat{cov}(Z, X)$. An estimate of the variance of $\widehat{\gamma}$ is $\widehat{\Sigma}_\gamma = \widehat{var}(Z) \otimes (\widehat{var}(X) - \widehat{cov}(Z, X)'\widehat{var}(X)^{-1}\widehat{cov}(Z, X))$. Then, a test of instrument relevance considers evidence against the null hypothesis $H_0 : \gamma = 0$, using the statistic

$$\widehat{W}_\gamma = (N - J)\widehat{\gamma}'\widehat{\Sigma}_\gamma^{-1}\widehat{\gamma}. \quad (3)$$

Note that by a central limit theorem and CramÃ©r's Theorem, since $\widehat{\Sigma}_\gamma \xrightarrow{p} var(Z) \otimes \sigma_V^2$, under $H_0 : \gamma = 0$, we have $\widehat{\Sigma}_\gamma^{-\frac{1}{2}}\sqrt{N - J}\widehat{\gamma} \xrightarrow{d} N(0, I_{J\times J})$ as $n \to \infty$. Hence, $\widehat{W}_\gamma \xrightarrow{d} \chi_J^2$ as $n \to \infty$.

The F-test statistic is defined as $\widehat{F} = \widehat{W}_\gamma / J$.

C: An R^2 measure under individual-level data

The sample R^2 in the model of Equation (2) is $\widehat{R}^2 = \widehat{\gamma}'\widehat{var}(Z)\widehat{\gamma}/\widehat{var}(X)$. The F-statistic $\widehat{F}$ can be written in terms of $\widehat{R}^2$,

$$\widehat{F} = \frac{N - J - 1}{J} \cdot \frac{\widehat{R}^2}{1 - \widehat{R}^2} \quad (4)$$

Also, from Equation (4), $J\widehat{F}(1 - \widehat{R}^2) = (N - J - 1)\widehat{R}^2$ so that $J\widehat{F} = (N - J - 1 + J\widehat{F})\widehat{R}^2$ and

$$\widehat{R}^2 = \frac{J\widehat{F}}{N - J - 1 + J\widehat{F}}. \quad (5)$$

D: Calculating F-statistics and R^2 with univariable summary data

Suppose we have access to:

1. signed genetic variant correlations between any two variants j_1 and j_2, denoted $\rho_{j_1 j_2} = \widehat{cor}(Z_{j_1}, Z_{j_2})$
2. beta-coefficients from a linear regression of X on Z_j, denoted $\widehat{\beta}_j = \widehat{var}(Z_j)^{-1}\widehat{cov}(Z_j, X)$
3. standard errors of $\widehat{\beta}_j$, denoted as the square root of $\widehat{\sigma}_j^2 = \left[\widehat{var}(X) - \widehat{\beta}_j^2\widehat{var}(Z_j)\right] \Big/ \left[N \cdot \widehat{var}(Z_j)\right]$
4. the sample size N.

Then, note that $a_j = N\widehat{\sigma}_j^2 + \widehat{\beta}_j^2 = \widehat{var}(Z_j)^{-1}\widehat{var}(X)$. Second, let $b_j = a_j^{-1}\widehat{\beta}_j = \widehat{var}(X)^{-1}\widehat{cov}(Z_j, X)$ so that $b = (b_1, \dots b_J)' = \widehat{cov}(Z, X)\widehat{var}(X)^{-1}$.

Next, let C be a $J \times J$ matrix with its (j_1, j_2)-th element given by $c_{j_1 j_2} = \rho_{j_1 j_2} a_{j_1}^{-\frac{1}{2}} a_{j_2}^{-\frac{1}{2}}$ $= \widehat{cor}(Z_{j_1}, Z_{j_2})\widehat{var}(Z_{j_1})^{\frac{1}{2}}\widehat{var}(Z_{j_2})^{\frac{1}{2}}\widehat{var}(X)^{-1} = \widehat{cov}(Z_{j_1}, Z_{j_2})\widehat{var}(X)^{-1}$. Therefore, $C = \widehat{var}(Z)\widehat{var}(X)^{-1}$. Then, $\widehat{\Gamma} = \widehat{var}(Z)^{-1}\widehat{cov}(Z, X)$, i.e. the beta coefficient from a *multivariable* linear regression of X on $Z = (Z_1, \dots, Z_J)'$ can be computed as

$$\widehat{\Gamma} = C^{-1}b$$

and the variance-covariance matrix of $\widehat{\Gamma}$, which is $\widehat{\Sigma}_\gamma = \widehat{var}(Z)^{-1} \cdot \left[\widehat{var}(X) - \widehat{cov}(Z, X)'\widehat{var}(Z)^{-1}\widehat{cov}(Z, X)\right]$ can be computed as

$$\widehat{\Sigma}_\gamma = C^{-1} \cdot \left[1 - b'C^{-1}b\right].$$

Using Equation (3), we can plug in our estimates $\widehat{\Gamma}$ and $\widehat{\Sigma}_\gamma$ into Equation (3) to calculate $\widehat{W}_\gamma$, and then just divide by J to calculate the $\widehat{F}$ statistic as $\widehat{F} = \widehat{W}_\gamma \big/ J$.

Finally, after calculating $\widehat{F}$, just plug-in the estimate into Equation (5) to get the $\widehat{R}^2$ estimate.

2. Extended simulation study I

In practice, we often do not know whether the true causal variant(s) are captured by the genotyping array. Indeed, it is likely that in many cases they are not directly genotyped and are only tagged by nearby variants. To approximate this situation in the single-causal-variant scenario described in the main text, we removed the causal variant along with all variants in linkage disequilibrium with it above a given threshold (pairwise r^2 > 0.95, or > 0.8, or > 0.4) from the dataset. We then assessed how well different variant selection strategies could recover the true genetic variance. Results for this simulation analysis are illustrated in Figure S2.

As expected, genetic predictors derived from all strategies explained less variance in the simulated exposure than that by the true causal variant, because the causal variant and its close proxies had been removed from the dataset (Figure S2). For strategies that require a tuning parameter, such as modified LD-pruning, COJO, and PCA, increasing the tuning parameter led to more variants in the gene region being incorporated into the genetic predictor, resulting in a greater proportion of variance explained (Figure S2). All variant selection strategies that incorporate multiple variants from the gene region explained more variance than the lead-variant-only approach, likely because these additional variants partially recover information about the removed causal variant. The advantage of these multi-variant strategies became increasingly apparent as variants with progressively lower correlation thresholds to the causal variant (from r^2 > 0.95 down to r^2 > 0.4) were removed, which further weakened the performance of the lead-variant-only approach (Figure S2 C).

3. Extended simulation study II

We further evaluated the performance of multi-variant strategies in the presence of horizontal pleiotropy. Specifically, in this simulation we considered a scenario in which two independent causal variants influence the exposure, and horizontal pleiotropy is introduced by allowing one of these causal variants to also influence the outcome. There is no actual causal effect of the exposure on the outcome. Therefore, any deviation of MR estimate from the null indicates bias. Data generating mechanism is shown below:

$$X_i = \sqrt{0.25}G_{i1} + \sqrt{0.15}G_{i2} + \varepsilon_{xi}$$

$$Y_i = \sqrt{0.05}G_{i2} + \varepsilon_{yi}$$

where: X_i: Simulated exposure for individual I,

Y_i: Simulated outcome for individual I,

G_{i1}: Standardized genotyping data for the first causal variant,

G_{i2}: Standardized genotyping data for the second causal variant,

$\varepsilon_{xi} \sim N(0, 0.6)$: Residual error term for exposure with variance being 60%,

$\varepsilon_{yi} \sim N(0, 0.95)$: Residual error term for outcome with variance being 95%.

This simulation experiment was repeated 100 times. Results for this simulation analysis are shown in the Figure S3.

As expected, the lead-variant-only approach produced an unbiased estimate, because the stronger causal variant has no horizontal pleiotropic effect. In contrast, all multi-variant strategies produced biased MR estimates, presumably because the genetic predictors derived from these strategies included the pleiotropic causal variant or variants correlated with it (Figure S3).

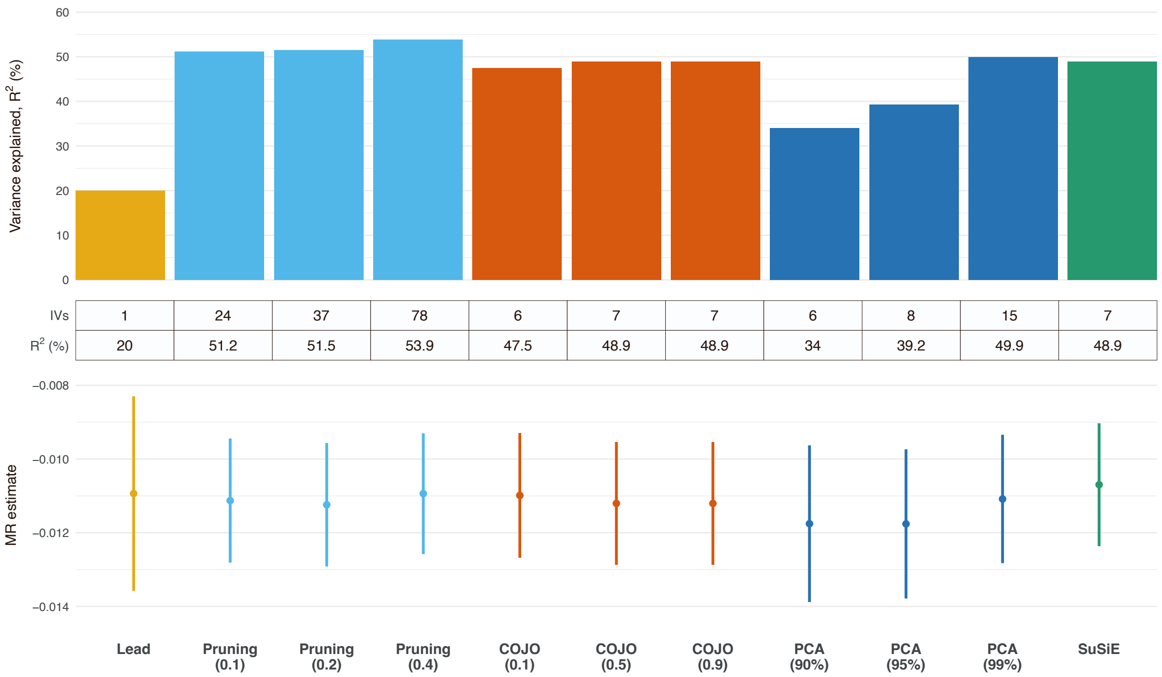

Figure S1: Proportion of variance in haptoglobin levels explained by variants selected using modified LD-pruning, COJO, SuSiE, and PCA, together with the corresponding MR estimates for the effect on red blood cell count, using the full set of variants in the haptoglobin region without p-value pre-filtering at the 0.001 threshold. The table below the plot shows the number of IVs and variance explained, R^2 (%). Error bars represent 95% CIs. For the modified LD-pruning, results are shown for pruning thresholds of r^2 = 0.1, 0.2 and 0.4. For PCA, results correspond to PCs capturing 90%, 95% and 99% of the variance in the LD matrix. For COJO, results are shown for collinearity thresholds of 0.1, 0.5, and 0.9.

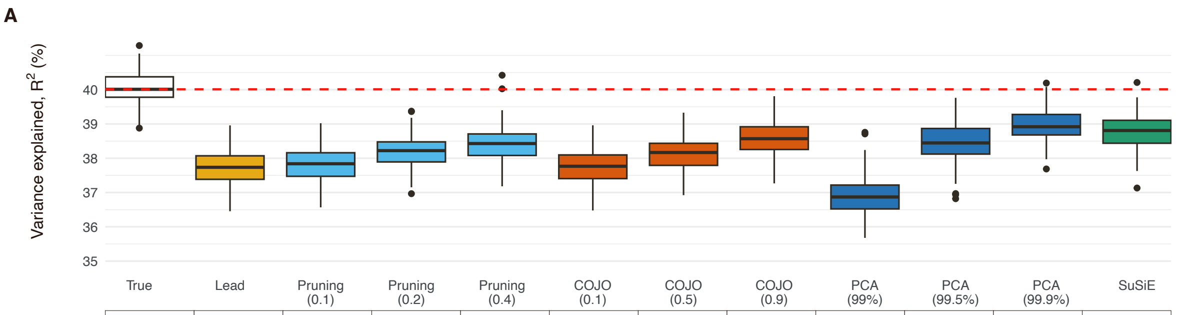

	True	Lead	Pruning (0.1)	Pruning (0.2)	Pruning (0.4)	COJO (0.1)	COJO (0.5)	COJO (0.9)	PCA (99%)	PCA (99.5%)	PCA (99.9%)	SuSiE
IVs	1	1	12	20	38.5	3	12	19	3	5	9	3
R^2 (%)	40	37.75	37.85	38.2	38.4	37.8	38.2	38.6	36.9	38.4	38.9	38.8

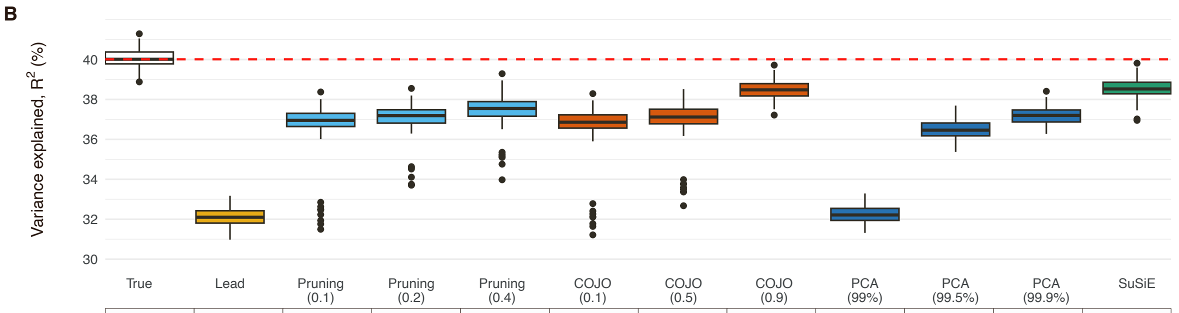

	True	Lead	Pruning (0.1)	Pruning (0.2)	Pruning (0.4)	COJO (0.1)	COJO (0.5)	COJO (0.9)	PCA (99%)	PCA (99.5%)	PCA (99.9%)	SuSiE
IVs	1	1	13	22	40	5	14	21	5	7	13	4
R^2 (%)	40	32.1	36.95	37.2	37.55	36.9	37.1	38.5	32.2	36.5	37.2	38.5

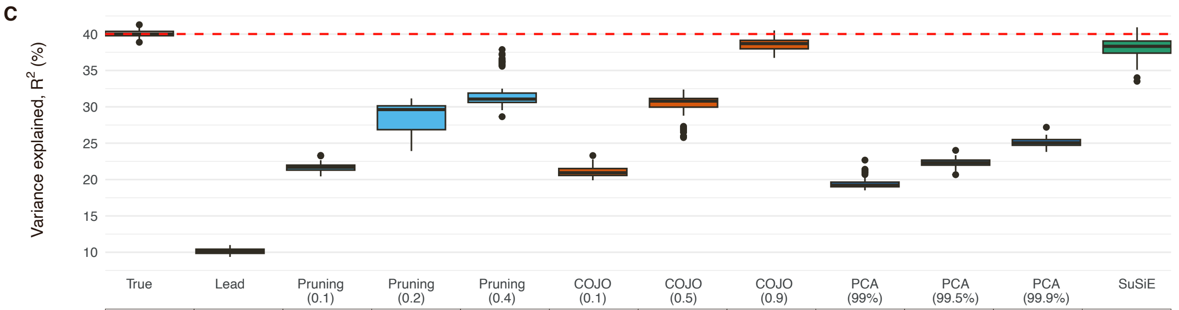

	True	Lead	Pruning (0.1)	Pruning (0.2)	Pruning (0.4)	COJO (0.1)	COJO (0.5)	COJO (0.9)	PCA (99%)	PCA (99.5%)	PCA (99.9%)	SuSiE
IVs	1	1	14	23	46	8	11	16	11	14	21	8
R^2 (%)	40	10.1	21.7	29.6	31.05	20.95	30.8	38.7	19.2	22.3	25	38.3

Figure S2: Proportion of variance in a simulated trait with one causal variant explained by variants selected using modified LD-pruning, COJO, SuSiE, and PCA, under scenarios where the causal SNP and its correlated SNPs are removed at three LD thresholds: (A) $r^2 > 0.95$, (B) $r^2 > 0.80$, and (C) $r^2 > 0.40$. Each simulation was repeated 100 times. The table below each plot shows the median number of IVs and median R^2 (%) across 100 replicates. The red dashed line indicates the median true R^2 (%), calculated from regressing the exposure on the true causal variant(s) using individual level data. Boxplots represent the estimates from each replicate. The box displays the lower quartile,

median, and upper quartile; whiskers extend to the minimum and maximum values within 1.5 x interquartile range from the lower and upper quartiles. Estimates outside this range are shown as individual points. For the modified LD-pruning, results are shown for pruning thresholds of r^2 = 0.1, 0.2 and 0.4. For PCA, results correspond to PCs capturing 99%, 99.5% and 99.9% of variance in the LD matrix. For COJO, results are shown for collinearity thresholds of 0.1, 0.5, and 0.9.

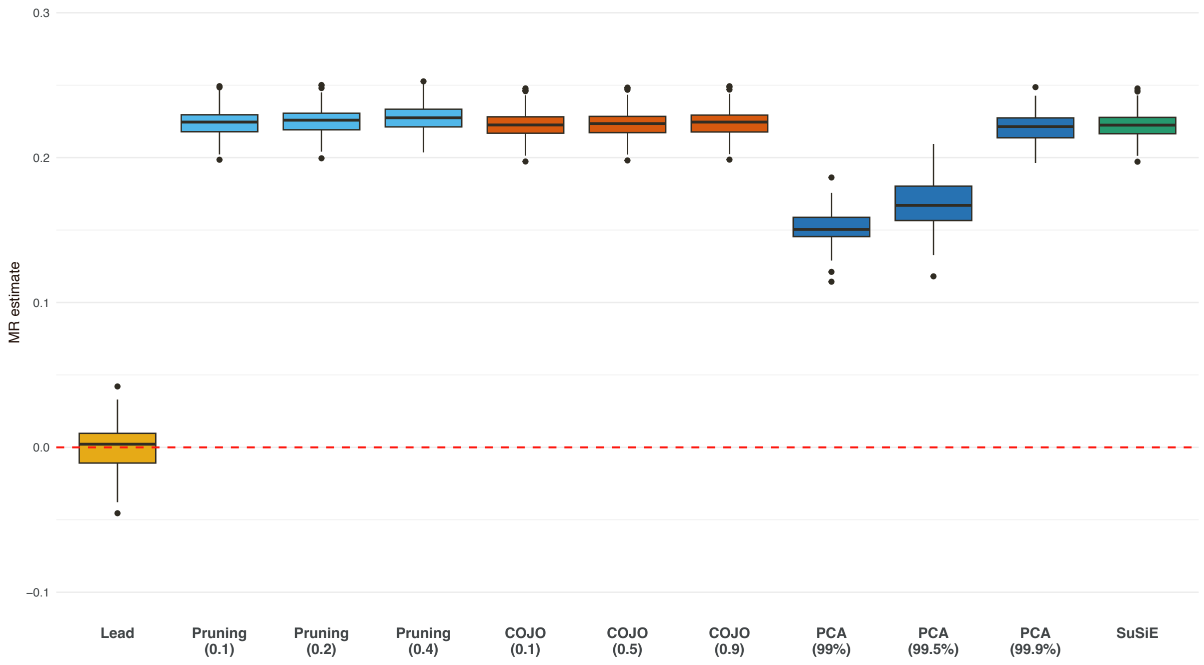

Figure S3: MR estimates obtained using variants selected by modified LD-pruning, COJO, SuSiE, and PCA in the simulation scenario with horizontal pleiotropy. Each simulation was repeated 100 times. The red dashed line indicates the target effect estimate. Boxplots represent the estimates from each replicate. The box displays the lower quartile, median, and upper quartile; whiskers extend to the minimum and maximum values within 1.5 x interquartile range from the lower and upper quartiles. Estimates outside this range are shown as individual points. For the modified LD-pruning, results are shown for pruning thresholds of r^2 = 0.1, 0.2 and 0.4. For PCA, results correspond to PCs capturing 99%, 99.5% and 99.9% of variance in the LD matrix. For COJO, results are shown for collinearity thresholds of 0.1, 0.5, and 0.9.
